# Annexin A2: the missing piece in the puzzle of pathogen-induced damage

**DOI:** 10.1080/21505594.2023.2237222

**Published:** 2023-07-23

**Authors:** Chao Li, Jianwei Yu, Daoyong Liao, Xiaoling Su, Xinchao Yi, Xue Yang, Jun He

**Affiliations:** a The Affiliated Nanhua Hospital, Department of Clinical Laboratory, Hengyang Medical School, University of South China, Hengyang, China; b Department of Public Health Laboratory Sciences, School of Public Health, Hengyang Medical School, University of South China, Hengyang, Hunan, China

**Keywords:** Annexin A2, S100A10, A2t, ligand, pathogen, therapeutic target

## Abstract

Annexin A2 is a Ca^2+^ regulated protein belonging to the Annexin family and is found in the cytoplasm and cell membrane. It can exist in a monomeric form or in a heterotetrameric form with the S100A10 dimer. The research on Annexin A2 in tumours is currently active, and studies on its role in pathogen infection are increasing. Annexin A2 plays a crucial role in the life cycle of viruses by mediating adhesion, internalization, uncoating, transport, and release. In the case of parasites, bacteria, mycoplasma, fungi, and other pathogens, Annexin A2 binds to the ligand on the pathogen, which mediates the pathogen’s adhesion to the host cell, ultimately leading to infection and damage to the host. Furthermore, some studies have developed biological or chemical drugs that target Annexin A2, which have demonstrated promising anti-infective effects. Thus, targeting Annexin A2 may present a promising therapeutic approach for the treatment of diverse infectious diseases. In summary, this paper provides an overview of Annexin A2 and its role in various pathogens. It highlights its regulation of pathogen infection and its potential as a therapeutic target for the treatment of infectious diseases.

## Introduction

**A**nnexin A2, also known as ANXA2, Annexin II, chromobindin VIII, calpactin I heavy chain, placental anticoagulant IV, lipocortin II, and p36, is a 38-kDa protein that belongs to a multigene family with over 160 members [1]. Annexin A2 is composed of the amino-terminal domain (N-terminal domain) and the carboxyl- terminal core domain (C-terminal core domain). The C-terminal core domain of Annexin A2 consists of four homologous domains tightly packed to form a curved disc with convex and concave surfaces [2,3]. The core convex surface domain contains phospholipid and Ca^2+^ binding sites [2,3]. The concave surface of Annexin A2 also contains the N-terminal and C-terminal regions associated multiple-binding sites. The N-terminal of Annexin A2 contains S100A10-binding site, the reactive cysteine residue and the phosphorylation sites of Tyr-23, Ser-11, Ser-25. Actually, the binding site of the molecular ligands of various viruses, bacteria and other pathogens is also in the N-terminal domain, which is the ligand-receptor binding site. And the C-terminal region contains heparin, F-actin, fibrin, RNA, negatively charged phospholipids and a reactive cysteine residue (Cys-334) binding sites [2–4]. It can exist in two forms: as a monomer molecule or as a heterotetramer A2t, which is composed of two Annexin A2 monomer molecules and a S100A10 dimer that are bridged by non-covalent bonds [1]. Its ligand, the dimer S100A10, has two binding pockets that can accommodate the N-terminus of Annexin A2 [5].

Due to the rising quantity of research on Annexin A2, its biological role is becoming more recognized. Intracellular Annexin A2 has been found to play a variety of roles in endocytosis, exocytosis, cellular apoptosis, cell proliferation and division, and inflammation [4]. Similarly, extracellular Annexin A2 also contributes to fibrinolysis and anticoagulation, cell metastasis, angiogenesis, phagocytosis [4]. Clinically, Annexin A2 has significant implications in the development of tumours, inflammation, and other diseases. It regulates tumour invasion, metastasis, and angiogenesis, among other things. For example, Annexin A2 plays a role in the homing and adhesion of prostate cancer cells to the bone marrow [6]. Additionally, Annexin A2 protein expression has manifested a favourable connection with the aggressiveness of tumours, resistance to anti-cancer drugs, shorter disease-free survival, and worse overall survival in clinical studies [7,8]. Furthermore, evidence shows that Annexin A2 is critical for endolysosomal membrane repair, and downregulation of Annexin A2 can impair endolysosomal membrane repair and activate the NLRP3 inflammasome, resulting in caspase activation and pro-inflammatory cytokine secretion in terms of inflammation [9].

Moreover, increasing evidence suggests that Annexin A2 serves as a crucial host protein for the successful invasion of microorganisms. Current findings indicate that Annexin A2 plays a constructive function in the virus life cycle, especially in virus adsorption, intracellular transport, and assembly (Table 1). Furthermore, Annexin A2 can facilitate infections caused by *Pseudomonas aeruginosa*, *Salmonella typhimurium*, *Escherichia coli*, and other pathogens by mediating bacterial adhesion and invasion [10]. Additionally, Annexin A2 is involved in mycoplasma-induced the pathogenicity of mycoplasma, tumour metastasis, and tumour drug resistance. Moreover, multiple Annexin A2 effects play a vital role in the transport of *Cryptococcus neoformans*, demonstrating Annexin A2’s distinctive role in the replication and infection of various viruses, pathogenic bacteria, mycoplasmas, parasites, and fungi. Therefore, this paper aims to review the critical role of Annexin A2 in pathogen infection and pathogenicity.

**Table 1.** The categories of virus proteins that interact with Annexin A2, the stages of the virus life cycle where Annexin A2 participates, and the locations of these interactions.

Virus^a^	Virus proteins	the stages of the virus life cycle	Action site
HPV	L2	Adsorption, cellular transport	Plasma membrane, cytoplasm
HIV	Gp120, gag(p55Gag, p41Gag)	Adsorption, assembly, maturation, and release	Plasma membrane, cytoplasm
HCV	NS5A, NS5B	Assembly, biosynthesis	DMVs
HBV	HBsAg, HBV pol	Adsorption, biosynthesis, celluar transport, and release	The whole cell
CMV	Glycoprotein B	adsorption	Plasma membrane
SADS-CoV	M protein	Assembly, release	Not available
PRRSV	N structural protein, Nsp9	Not available	cytoplasm
AIV	NS1	Not available	cytoplasm
MV	M protein	assembly	Plasma membrane
**Virus**^**a**^	**Virus proteins**	**the stages of the virus life cycle**	**Action site**
EMCV	2A	Not available	Plasma membrane, cytoplasm
EV71	VP1	adsorption	Plasma membrane

Note: ^a^HPV, *human papillomavirus*; HIV, *human immunodeficiency virus*; HCV, *hepatitis C virus*; HBV, *hepatitis B virus*; CMV, *cytomegalovirus*; SADS-CoV, *swine acute diarrhoea syndrome coronavirus*; PRRSV, *porcine reproductive and respiratory syndrome virus*; AIV, *avian influenza virus*; MV, *measles virus*; EMCV, *encephalomyocarditis virus*; EV71, *enterovirus 71*.

## Human papillomavirus

*Human papillomavirus* (HPV) is known to cause hyperplastic lesions in human skin and mucous membranes. Chronic infection with HPV has been linked to the development of cervical cancer, vaginal cancer, anal cancer, oropharyngeal cancer, and penile cancer [11]. Studies have shown that Annexin A2 is an important factor for successful HPV infection of host cells. Furthermore, the virus can also use Annexin A2 to evade or suppress the host immune response.

### Role of internalization and intracellular transport of HPV

**Figure 1.**
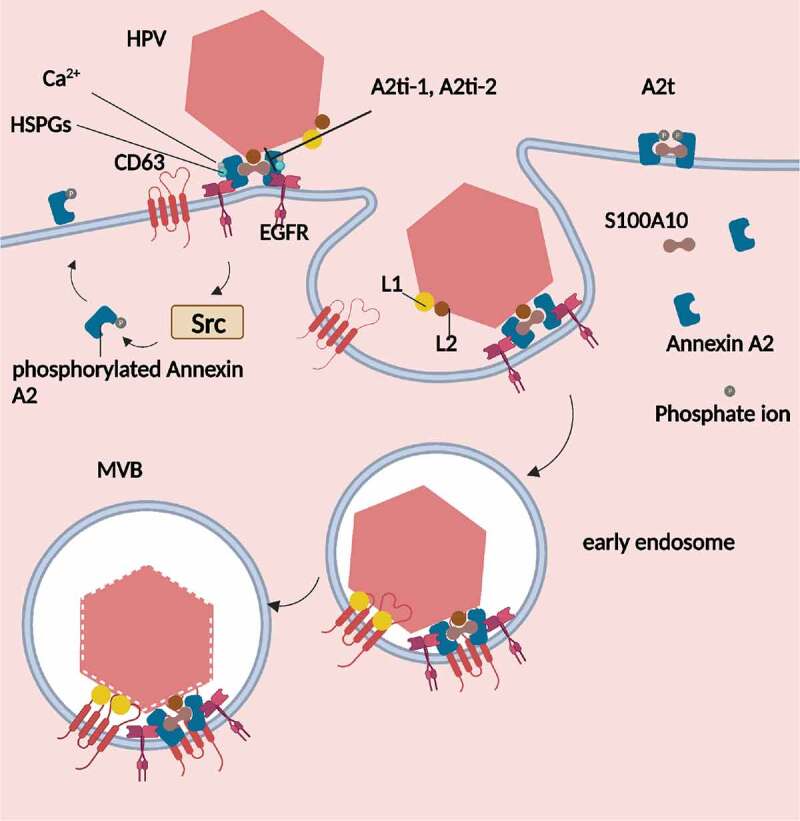
Annexin A2 plays a crucial role in mediating both the adhesion and transport of HPV virions. Upon infecting epithelial cells, Annexin A2 is phosphorylated and moves outside the cell. When HPV adheres to the cells, S100A10 in the A2t heterotetramer binds to the minor capsid protein L2, facilitating the process of HPV endocytosis. During transport, the major capsid protein L1 binds to CD63, and A2t binds to CD63, promoting the movement of HPV from early vesicles to multivesicular bodies.

Numerous studies have demonstrated that Annexin A2 and its heterotetrameric A2t play a crucial role in the internalization and intracellular transport of HPV (Figure 1). Upon exposure of HPV16 to HaCat cells, a signalling cascade, the EGFR-dependent Src protein kinase activation process is triggered that results in the phosphorylation of Tyr23 of Annexin A2. This phosphorylation event leads to the translocation of Annexin A2 to the outer surface of the plasma membrane [12]. At this point, S100A10 dimer forms a heterotetramer with Annexin A2. HPV16 then binds to A2t on the plasma membrane in a manner that is dependent on Ca^2+^ and heparan sulphate proteoglycans (HSPGs), thereby adhering to the cells [12,13].

It is important to note that HPV is a double-stranded DNA and non-enveloped virus, and its capsid contains two proteins, major capsid protein L1 and minor capsid protein L2 [14]. When HPV adheres to the cell surface, the amino acids 108–126 of the minor capsid protein L2 specifically bind to the subunit S100A10 of A2t on the membrane of host cells, with an association constant (k) of 10^5^ M^−1^ [13]. Additionally, HPV16 L1L2 VLP (virus-like particle) invades Langerhans cells more than twice as much as HPV16 L1 VLP [15].

After HPV invades the host cells, it is transported through vesicles within the host cell. The process of HPV post-invasion transport to the multivesicular body (MVE) and subsequent capsid cleavage depends on the presence of CD63, the MVE biomarker [16]. Annexin A2 binds to CD63, promoting the transport of HPV from the early endosome to the multivesicular endosome. Annexin A2 also protects HPV from degradation by more acidic lysosomes and inhibits HPV capsid cleavage [17]. Previous research has shown that CD63 binds to HPV L1 [16], suggesting that Annexin A2 on the host vesicle membrane may also indirectly bind to L1 through CD63, promoting intracellular transportation of HPV. Further research is needed to investigate this possibility.

After HPV invades cells, it is suggested that HPV induces pathological changes with the help of Annexin A2. HPV16 contains an oncogenic protein called E5, which is embedded in the endoplasmic reticulum and nuclear membrane, with its C-terminal exposed to the cytoplasm. E5 binds to Annexin A2 and promotes the redistribution of Annexin A2 and its ligand S100A10, targeting these molecules to the perinuclear region. This process promotes the fusion of the perinuclear membrane and induces cell vacuolation [18,19].

### Annexin A2 helps HPV inhibit host immune response

When HPV infects Langerhans cells (LC), researches shows that the exogenous A2T complex decreases the secretion of TH1-related cytokines and the expression of MHC II and CD86 on the surface of the LC [20]. This complex can also block the PI3K-AKT signaling cascade pathway of LC. Similarly, inhibiting the endogenous A2t molecule using the small molecule A2t inhibitor, A2ti-1, can boost the immune response to HPV16 [20]. Annexin A2 and S100A10 form a compound molecule A2t, which can combine with L2 and promote the internalization of HPV [15,20]. When HPV16 L1 virus-like particles (VLPs) are exposed to LC, the migration of LC is enhanced, and cytokines and chemokines synthesized by Th1 cells are highly secreted compared to the uninfected group. These cytokines and chemokines include TNF-α, IL-12p70, IL-6, IL-8, IFN-inducible protein 10, MCP-1, MIP-1β, and RAN-TES. Additionally, the CD8+ T cell immune response is also reinforced [15]. However, these phenomena are not obvious after exposure to HPV16 L1L2 VLP, which suggests that L2 helps HPV inhibit the immune response [15]. Specifically, L2 inhibits the PI3K-AKT pathway, which is related to the immune response [15]. Therefore, based on the above findings, it is speculated that Annexin A2 and L2 together may help HPV escape host immunity by blocking the PI3K-AKT pathway.

### A2t inhibitors reduce HPV infection

The A2t inhibitors (A2ti) were found to significantly inhibit the interaction between Annexin A2 and S100A10. Previous studies have suggested that 1,2,4-triazole compounds may serve as inhibitors of the Annexin A2-S100A10 protein interaction through three-dimensional pharmacophore design and biochemical screening tests. These compounds possess a common acetylation side chain and benzene ring, which may inhibit the binding of Annexin A2 and S100A10 [21]. Woodham et al. applied this finding to control HPV infection and demonstrated that two A2ti compounds, A2ti-1:2-[4-(2-ethylphenyl)-5-o-tolyloxymethyl-4 H- [1,2,4]triazol-3-ylsulfanyl]acetamide and A2ti-2:2-(4-phenyl-5-o-tolyloxymethyl-4 H [1,2,4]triazol-3-ylsulfanyl)acetamide, effectively inhibited HPV infection in vitro in epithelial cells [22]. Notably, A2ti-1 demonstrated stronger inhibition of HPV infection and internalization comparedto A2ti-2. Isothermal titration calorimetry confirmed that both A2ti compounds targeted the A2T subunit S100A10, thus preventing HPV pseudovirus entry into cells [22]. These A2ti compounds were found to specifically inhibit HPV infection without affecting other viruses such as HIV.

In summary, the research suggests that the A2ti compounds can inhibit the Annexin A2-S100A10 protein interaction, thereby inhibiting HPV infection. Specifically, A2ti-1 was found to be more potent than A2ti-2 in preventing HPV infection in epithelial cells. The mechanism of action appears to be related to the compounds’ ability to target the A2T subunit S100A10. Importantly, these compounds were found to have specificity towards HPV and did not affect other viruses.

## Human immunodeficiency virus

**Figure 2.**
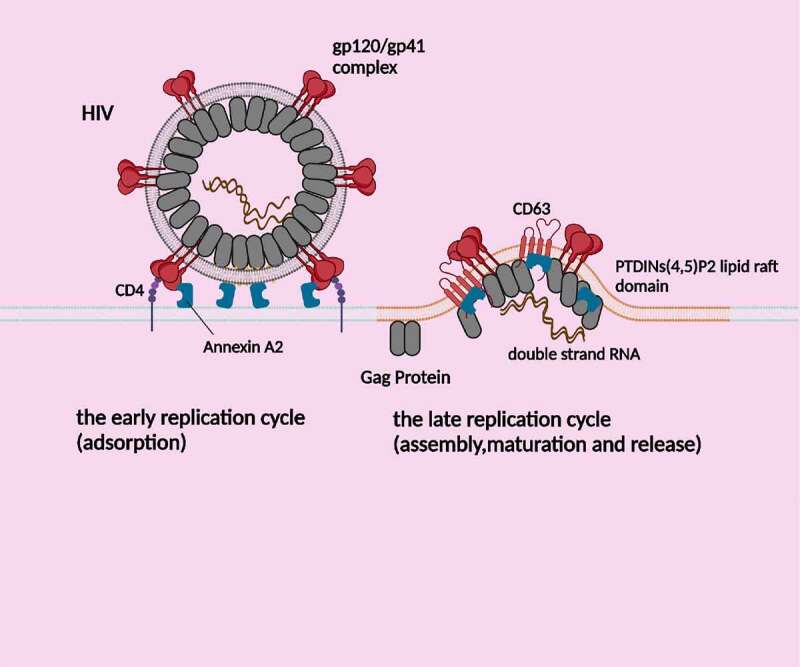
Annexin A2 mediates adhesion and late replication cycle of HIV. (1) Annexin A2 assisted HIV gp120 to bind to host cell CD4 molecule; (2) Gag proteins target to the lipid raft microdomain rich in PTDIN (4,5) P2, binds to Annexin A2, and then interacts with CD63 to mediate virus assembly, maturation and release.

Human infection with HIV can lead to T-cell damage, reduced cellular immune function, and various forms of organ damage such as chronic cardiovascular disease, liver disease, lung disease, and central neurological diseases and disorders [23,24]. Annexin A2 plays a crucial role in HIV infection by aiding in the virus’s absorption, assembly, maturation, and budding. This protein is an assistant molecule for HIV-1 infected macrophages. Blocking Annexin A2 with anti-Annexin A2 antibodies or siRNA interference significantly reduces HIV infection by measuring the decreasing of p24Gag and other indicators [25–27]. HIV-1 is a RNA virus with an envelope, and the lipid and protein on its envelope combine with Annexin A2 to facilitate HIV infection of host cells. These components include phosphatidylserine (PS), gp120, p55Gag, among others (Figure 2).

### Role of adsorption, assembly, maturation and release in the HIV replication cycle

During the adsorption phase of HIV, PS on the virus’s outer envelope binds directly to Annexin A2, facilitating its infection of monocyte-derived macrophages (MDMs). However, secretory leukocyte protease inhibitor (SLPI) can disrupt this binding [25]. While Woodham and his team demonstrated an indirect and unstable binding between Annexin A2 and HIV-1 gp120 in vitro [28], the specific binding site and function remain unclear. As is well-known, during the HIV adsorption and fusion process, the envelope protein gp120 of HIV first binds to CD4 molecule on the surface of target cells. Annexin A2 also has the capacity to bind to gp120, suggesting that Annexin A2 may play an auxiliary receptor role in the process of gp120 binding to CD4.

During the late phase of HIV-1’s replication cycle, the virus preferentially assembles and buds from lipid raft membrane microdomains. Its structural protein p41Gag and Annexin A2 directly bind to the phosphatidylinositol (4,5) diphosphate [PTDlNs(4,5)P2] lipid raft membrane domain of the cell membrane of 293T cells [29]. Previous studies have also shown that Annexin A2 binds directly to p55Gag and CD63, mediating viral assembly and release [26]. Gag targeting to lipid rafts is thought to be modulated by PTDlNs(4,5)P2, a signal phospholipid, whose depletion results in Gag redistribution to CD63-positive late endosomes or multivesicular bodies (MVBs) in cells supporting HIV budding from the plasma membrane [30]. In macrophages, siRNA-mediated decrease of Annexin A2 expression resulted in incomplete processing of gag protein and ineffective incorporation of CD63 into viral particles, both of which are important steps in viral maturation and assembly [26]. Surprisingly, Annexin A2, as a PTDlNs(4,5)P2-binding protein, is suggested to promote the construction of lipid microdomains required for exocytosis. HIV TAT protein expressed in pheochromocytoma PC12 cells displaces Annexin A2 from the periphery of stimulated cells. The high affinity of TAT for PTDlNs(4,5)P2 interferes with the secretion-dependent recruitment of Annexin A2 to the cell membrane, affecting cell secretion [31]. It is worth investigating whether this represents a negative feedback mechanism for HIV hiding in cells.

### Analysis of Annexin A2 target therapy for HIV

SLPI is a protein that disrupts the binding of PS to Annexin A2, effectively preventing HIV infection of macrophages [25]. Woodham et al. demonstrated that an antibody against the N-terminal of Annexin A2 suppressed the production of HIV-1 particles by macrophages, similar to the anti-HIV drugmalaviroc [28]. However, the emergence of HIV-1 resistant strains often occurs with inhibitors of HIV entry like maraviroc [32,33]. To study other options, Woodham’s team tested three different drugs targeting Annexin A2 and found that none of them decreased viral load [28], but A2ti-1 and A2ti-2 effectively inhibited HPV internalization. This suggests that Annexin A2 may promote the HIV-1 infection of the macrophage in monomeric form without the involvement of S100A10 dimer, which differs from the mechanism of HPV infection that involves the heterotetrameric molecule of Annexin A2. Therefore, SLPI-related drugs and antibodies against Annexin A2 monomers could be developed for clinical treatment of HIV infection, and chemical drugs against Annexin monomers are urgently needed.

## Hepatitis C virus

*Hepatitis C virus* (HCV) infection is a main factor causing severe liver disease, such as chronic hepatitis, liver cirrhosis, hepatocellular carcinoma etc.; according to the data of World Health Organization (WHO), about 71 million people are now infected with HCV and at least 400,000 people died from HCV disease every year [34].

### Role of assembly of HCV

**Figure 3.**
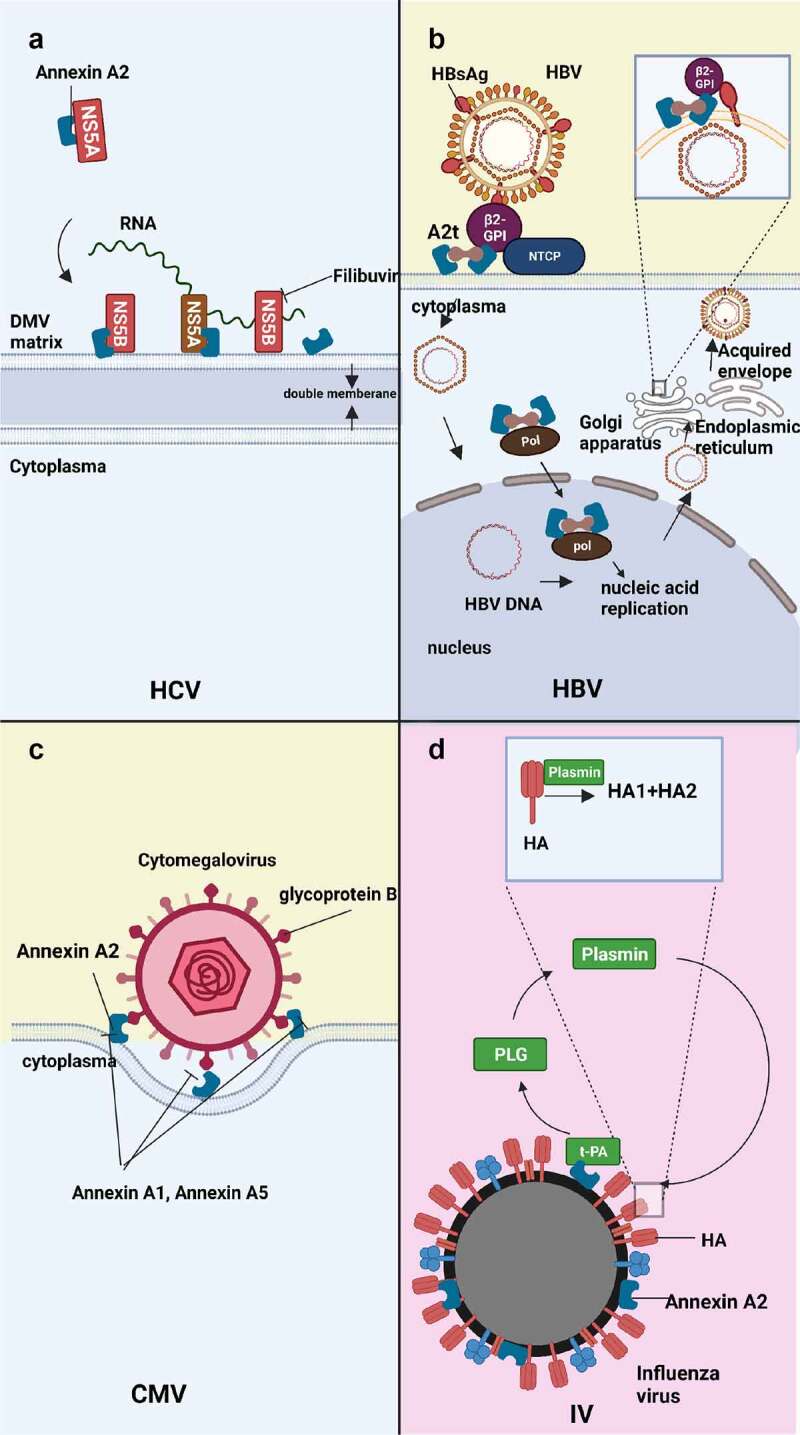
Effect of Annexin A2 in HCV, HBV, CMV, influenza virus infection. (a) Annexin A2 mediates HCV assembly in DMV. Annexin A2 monomer binds to NS5A and NS5B in the lipid-rich membrane microenvironment of DMV to promote virus assembly and replication. (b) Annexin A2 mediates HBV Adhesion, Nucleic Acid Replication and Assembly, budding. In the adhesion stage, Annexin A2, β2-GPI, NTCP, and HBsAg form a complex that facilitates the fusion of the viral envelope with the host cell envelope. During nucleic acid replication, Annexin A2 transports HBV polymerase to the nucleus. In the budding stage, the HBV nucleocapsid acquires the envelope from the Golgi apparatus or endoplasmic reticulum. HBsAg, β2-GPI, and Annexin A2 bind to the envelope and facilitate HBV budding. (c) Annexin A2 mediates CMV adhesion. During the process of adhesion, the glycoprotein B located on the envelope of CMV interacts with Annexin A2 located on the membrane of the host cell. This interaction leads to the binding of the two surfaces, facilitating the attachment of the virus to the host cell. (D) Annexin A2 mediates the replication of influenza virus. According to the mainstream theory, the haemagglutinin (HA) protein of the influenza virus undergoes cleavage into two parts, HA1 and HA2, which are involved in the process of virus replication. Annexin A2 plays a crucial role in this process by binding to t-PA, and the resulting complex facilitates the conversion of PLG into plasmin, which in turn assists in the cleavage process.

Recent research has demonstrated that Annexin A2 binds with non-structural proteins NS5A and NS5B to facilitate viral assembly (Figure 3a) and may also play a role in viral RNA replication during HCV infection. Knocking down Annexin A2 does not obviously impact HCV RNA production, but it does lead to a considerable reduction in both extra- and intracellular virus titres [35,36]. Therefore, it was concluded that Annexin A2 likely influences the assembly rather than the replication and release of HCV virions. NS5B is a RNA-dependent RNA polymerase of HCV, and its polymerase activity is decreased when in complex with Annexin A2, indicating that Annexin A2 is not involved in events requiring an active NS5B polymerase in the viral life cycle [37]. These findings support the hypothesis that Annexin A2 plays a role in HCV replication complex assembly rather than in genome replication.

In addition, recent studies also have shown that domain III of NS5A, a nonstructural protein, is crucial for HCV assembly, but not for HCV RNA replication [38–40]. This protein recruits Annexin A2 to the HCV replication sites located in the membrane of double-membrane vesicles (DMVs), which are membranous web structures [35]. Colocalization analysis has revealed that NS5A is involved in the recruitment of Annexin A2, although no evidence showed direct interaction between the two proteins [35]. But afterwards, Saxena et al. have demonstrated that HCV nonstructural proteins, including NS5A, directly interact with Annexin A2 on the DMV membrane, which potentially helps to recruit various phosphatidylinositides such as PI4P to create a lipid-enriched membrane microenvironment where viral replication complexes can assemble [41]. However, they have also found that the siRNA-mediated knockdown of Annexin A2 leads to a significant decrease in viral proteins, viral RNA, and replicase activity in vitro, suggesting that Annexin A2 may play a role in HCV RNA replication [41]. It is worth noting that these findings contradict Backes’ research, which may be attributed to differences in the siRNA targeting Annexin A2. Therefore, the precise role of Annexin A2 in HCV replication is still an active area of investigation.

Besides, during extracellular transport of HCV RNA encapsulated in exosomes, knockdown of Annexin A2 in SGR cells reduced the ability of co-cultured dendritic cells to produce IFN-α which can deal with viral infections by more than 20 times [36]. This suggests that Annexin A2 may aid in host innate immunity to HCV. Therefore, Annexin A2 is a double-edged sword for HCV. It can not only promote HCV assembly, but also promote the body’s innate immunity to HCV. As a result, the development of drugs or antibodies targeting Annexin A2 that can balance the relationship between the two above could be a promising avenue for future research.

### Analysis of Annexin A2 target therapy for HCV

NS5B is a crucial 68 kDa RNA polymerase that plays a central role in HCV replication and is often targeted by HCV drugs. One such drug is Filibuvir (PF-868554), which is being developed by Pfizer as an orally-administered, non-nucleoside inhibitor of the HCV NS5B RNA-dependent RNA polymerase for the potential treatment of chronic HCV infection [42]. Recent findings by Solbak et al. [37] have shown that the interaction between Annexin A2 and NS5B interferes with the ability of NS5B to interact with the allosteric inhibitor Filibuvir significantly. This interaction also impairs the polymerase activity of NS5B, which is evident from the significantly reduced nucleotide incorporation rate. Annexin A2 appears to be necessary for viral replication to occur at the correct location. From a HCV drug discovery perspective, novel drugs may be designed to target the Annexin A2-NS5B interaction interface, which could increase the efficacy of Filibuvir and reduce the binding of Annexin A2 to NS5B.

## Hepatitis B virus

The *hepatitis B virus* is responsible for causing acute and chronic hepatitis, which can increase the risk of death from cirrhosis and liver cancer. The pathogenesis ofHBV is complex, and studies have shown that Annexin A2 plays an auxiliary role in various steps of the HBV life cycle in infected cells, such as adsorption, nucleic acid replication, intracellular transport, and release (Figure 3b). Annexin A2 serves as the receptor of β2GPI on the membrane, and β2-GPI can bind to HBsAg [43]. β2GPI, also known as apolipoprotein H, is a human plasmatic protein primarily synthesized in the liver. High expression of β2-GPI facilitates the binding of HBsAg to cell surfaces, which is essential for the transfer of HBV virus particles to the sodium taurocholate co-transporting polypeptide (NTCP) receptor and interaction with Annexin A2 for viral membrane fusion [44]. Thus, Annexin A2 is thought to be involved in HBV adsorption and membrane fusion. HBV polymerase interacts with S100A10(p11), a Ca^2+^ modulated protein previously shown to bind to Annexin A2 [45]. Immunofluorescence analysis demonstrates that p11 recruits HBV polymerase to promyelocytic leukaemia nuclear bodies (PMLNBs) [45]. Association of HBV polymerase and p11 with PML NBs may help the viral genome for initiation of nucleic acid replication and transcription. In terms of virus budding, HBV acquires its envelope directly from the endoplasmic reticulum or Golgi apparatus to form secretory vesicles. Recent studies [46] have shown that A2t is involved in the process of HBV exocytosis in trophoblasts. Annexin A2 is transferred from the cytoplasm to the surface of autophagosomes containing HBV and recruits S100A10 to form the heterotetramer A2t. A2t then recruits VAMP2, SNAP25 for membrane fusion, and the autophagosome fuses with MVB. HBV is translocated across the trophoblast via A2t and MVB mediated exocytosis.

It is assumed that HBV adsorption, polymerase recruitment, transport and release require A2t rather than Annexin A2 or S100A10 molecules alone. HBV is released from the endoplasmic reticulum and Golgi apparatus and obtains the envelope. And the β2-GPI, Annexin A2 and HBsAg are mainly colocalized in the cytoplasm, so it is inferred that these molecules bind heavily on the above two organelles.

Regarding HBV-induced liver tumours, the promoter methylation or binding of Annexin A2 gene by HBV oncogene influences the occurrence and development of liver cancer. HBV is involved in epigenetic regulation by methylation of the promoter of the Annexin A2 gene, which inhibits the expression of the Annexin A2 protein in HepG2.2.15 cells [47]. Additionally, in HepG2 cells, Annexin A2 significantly inhibits the secretion of HBsAg and increases the intracellular storage of HBsAg, which is closely related to the occurrence of liver cancer [48]. Thus, it is speculated that the methylation of Annexin A2 gene promoter in liver cells may inhibit Annexin A2 expression, reduce the storage of HBsAg, and decrease the risk of liver cancer, which may be the body’s own defence mechanism. Moreover, in HBV-associated liver hepatocellular carcinoma (LIHC), the ETV4 oncogene binds to the Annexin A2 promoter, enhancing the expression of Annexin A2 at the level of transcription. This process promotes the activation of the Wnt-beta-catenin pathway, inducing hepatocellular carcinoma cell migration and proliferation, and promoting the progression of HBV-associated LIHC [49]. Blocking the binding of Annexin A2 and ETV4 may slow down the progression of liver cancer.

## Cytomegalovirus

*Cytomegalovirus* (CMV) is a significant pathogen that causes severe diseases in immunocompromised individuals, including transplant patients, those with AIDS, and immunologically immature newborns [50]. The process of CMV causing disease involves its adsorption, during which Annexin A2 may play a helpful role (Figure 3c). In 1994, Wright et al. identified Annexin A2, an about 36 kDa protein, on the surface of human umbilical vein cells, which binds to CMV [51]. Moreover, this protein interacts with CMV surface anionic phospholipids in a calcium-dependent manner [51,52]. Through a model using a synthetic phospholipid membrane containing anionic phospholipids and a mixtureof Annexin A2 monomers and heterotetramers, it was demonstrated that Annexin A2 plays a bridging role in CMV and phospholipid membrane association [53]. Envelope glycoprotein Bon CMV can physically bind to Annexin A2 on the surface of human foreskin fibroblasts in a calcium-independent manner [54]. Based on this knowledge, it can be speculated that Annexin A2 is a host receptor for the *cytomegalovirus* envelope glycoprotein. Additionally, Annexin A1 and Annexin A5 bind to Annexin A2 and inhibit CMV infection of host cells and fusion with the host cell membrane [53,55–57]. Currently, there are limited therapeutic options against cytomegalovirus. Developing Annexin A2 drugs or antibodies on the host side, combined with existing drugs, may be helpful to treat with CMV infection.

## Nidovirus

The nidovirus order is a group of positive-stranded RNA viruses, which includes four families. Some well-known human pathogens, such as *MERS-* and *SARS-coronavirus* (SARS-CoV), as well as economically important animal nidoviruses, such as *porcine reproductive and respiratory syndrome virus* (PRRSV), *porcine epidemic diarrhoea virus* (PEDV), *equine arteritis virus* (EAV), and *chicken infectious bronchitis virus* (IBV), belong to this order. Due to the significant morbidity and mortality associated with nidoviruses, there has been extensive research on the virus in recent years. Regarding Annexin A2, Researchers have primarily focused on autoimmune diseases and the interaction between nidoviruses and Annexin A2 to develop broad-range antiviral agents.

### Annexin A2 and coronavirus infections

Coronaviruses typically infect the upper respiratory and gastrointestinal tracts of mammals and birds, resulting in colds and pneumonia in humans and respiratory and digestive diseases in animals. The discovery of the human coronavirus SARS-CoV, which causes severe acute respiratory syndrome (SARS), has led to increased attention on coronaviruses in the research community. Recent research progress on the interaction between Annexin A2 and coronaviruses is summarized below.

SARS-CoV-induced pathological damage may be associated with autoimmunity. Annexin A2, a molecule involved in coronavirus infection, may act as an autoantigen. Antibodies against the SARS-CoV spike-protein domain 2 (S2) in the sera of SARS patients are partly responsible for the cross-reactivity with epithelial cells [58,59]. Cytokines IL-6 and IFN-γ induced by SARS-CoV increase the expression of Annexin A2 in lung epithelial cells, which enhances the cross-reactivity of autoantibodies against SARS-CoV S2 and epithelial cells [60]. Annexin A2 is identified as the autoantigen of the antibody, which may be one of the reasons why SARS-CoV causes lung damage in patients. Similarly, recent investigations have found the presence of anti-Annexin A2 autoantibodies in hospitalized COVID-19 patients, with higher serum concentrations in patients who died from COVID-19 than in survivors [61–63]. The level of lung involvement, as measured by chest CT, was also found to be increased in COVID-19 patients with higher serum Annexin A2 levels [64]. Although it is unclear whether Annexin A2 is involved in the life cycle activities of SARS-CoV and SARS-CoV-2, reducing the levels of autoantibodies is urgent.

Annexin A2 has been also found to play a part in the life cycle of various animal coronaviruses, including *swine acute diarrhea syndrome coronavirus* (SADS-CoV) and *infectious bronchitis virus* (IBV). The membrane (M) protein, participating in virus assembly and budding, is the most abundant structural protein in coronaviruses, and SADS-CoV membrane (M) protein interacts with Annexin A2 in Vero cells and IPI-2I cells [65,66]. Furthermore, Kwak et al. demonstrated that Annexin A2 binds to IBV mRNA pseudoknots, and that knockout of Annexin A2 significantly increases the IBV mRNA frameshift efficiency, thereby enhancing translation efficiency [67]. Given that viral frameshifting is critical for many pathogenic viruses, including coronaviruses, the interaction between Annexin A2 and IBV mRNA pseudoknots may provide a novel target for antiviral drug discovery [68,69].

In conclusion, Annexin A2 plays a crucial role in various aspects of coronavirus infection. While it can have protective effects such as reducing the frameshift efficiency of the virus, it can also have detrimental effects such as promoting virus assembly and budding, and serving as an autoantigen that contributes to host tissue damage. The dual nature of Annexin A2 in coronavirus infection highlights its complexity and underscores the need for further investigation to elucidate its exact role in the viral life cycle and to identify potential targets for antiviral therapies.

### Annexin A2 and arterivirus infections

Arteriviruses, which include *equine arteritis virus*, *porcine reproductive and respiratory syndrome virus* (PRRSV), *simian hemorrhagic fever virus*, and *lactate dehydrogenase-elevating virus* (LDV) of mice, have been found to interact with Annexin A2 in various ways [70]. Specifically, the B domain (109-174AA) of Annexin A2 binds to vimentin, a cytoskeletal component, and the Annexin A2-vimentin complex then binds to the N structural protein of PRRSV, which may promote virus infection [71]. Additionally, one study has suggested that Annexin A2 is closely associated with RNA replication of arteriviruses and gets incorporated into PRRSV particles in infected cells [72]. Annexin A2-Nsp9 interaction has also been detected both in vitro and in vivo of PRRSV-infected cells, which suggests that Annexin A2 may affect viral RNA synthesis and virus replication [73]. Interestingly, the knockout of Annexin A2 significantly reduced the viral and RNA copy numbers of progeny, indicating the importance of Annexin A2 in PRRSV infection [73]. Furthermore, Liu et al. [74] established a pig lung transplantation model of PRRSV infection in immunodeficient mice and successfully used this model to verify that the CXCR3 antagonist AMG487 effectively inhibited PRRSV infection and alleviated lung injury by down-regulating the expression of Annexin A2. It is worth considering whether CXCR3 antagonists can be used in the treatment of patients with COVID-19 to decrease the concentration of Annexin A2.

The precise mechanism by which Annexin A2 facilitates nidovirus replication and infection remains unclear, and the specific interactions between Annexin A2 and nidovirus proteins warrant further investigation.

## Influenza virus

*Influenza A virus* and avian influenza virus are highly pathogenic strains of influenza that are associated with high morbidity and mortality. Recent studies have shown that Annexin A2 plays a role in influenza virus replication by affecting the transformation of plasminogen (PLG) (Figure 3d). The cleavage of haemagglutinin molecules into HA1 and HA2 subunits by trypsinoids is necessary for the infection of target cells by *influenza A virus* [75]. PLG is converted to plasmin with trypsin-like protease activity, which can compensate for the deficiency of trypsin in mice [76,77]. Annexin A2 contributes to fibrinolysis by binding to t-PA, thereby facilitating plasmin production [78,79]. The protein of *H1N1*, *H3N2*, and *H6N2* influenza viruses binds to PLG and promotes its conversion to plasmin [80]. Similarly, Su et al. [81] found that Annexin A2 binds to PLG and mediates its conversion to plasmin, which enhances the replication ability of *avian influenza virus*(AIV) *H9N2* SH7 and plays an important role in its airborne transmission. Targeting Annexin A2 may be a potential treatment for influenza virus infections.

However, the replication of *avian influenza virus* (AIV) is also regulated by non-structural protein 1 (NS1). Annexin A2 has been shown to interact with AIV H5N1 NS1 and promote virus proliferation both in vitro and in vivo. This interaction leads to a significant increase in the titre and expression of AIV proteins (HA andM1) [82]. Interestingly, the mechanism behind this interaction does not require the conversion of PLG to plasmin to initiate H5N1 infection. Instead, Annexin A2 can directly interact with influenza viruses to mediate infection without using the fibrinolytic conversion mechanism. 

## Other viruses

Annexin A2 is a host protein that is helpful to the replication and infection of some viruses, such as *measles virus* (MV), *enterovirus 71* (EV71), and *encephalomyocarditis virus* (EMCV), by interacting with viral proteins (Table 1). Specifically, the interaction between Annexin A2 and the N-terminal region of the M protein of MV is essential for the localization of the M protein on the plasma membrane, which connects the nucleocapsid and the envelope protein, facilitating viral assembly [83]. It is hypothesized that Annexin A2 may facilitate the transport of M protein to the plasma membrane and the localization of the M protein-bound RNP complex on the plasma membrane. Additionally, the virulence protein 2A of EMCV interacts with Annexin A2 via the activation of the JNK/c-Jun pathway, which inhibits apoptosis and promotes EMCV replication during the early stage of infection [84]. Similarly, Annexin A2 promotes the adhesion of EV71 to host cells by binding to the capsid protein VP1 [85]. Furthermore, Annexin A2, PI4KB, and 3D polymerase work together to reshape cell membranes and form replicating organelles for EV71 [86]. Finally, Annexin A2 also protects against intracerebral microhemorrhages caused by Ebola virus infection by stabilizing the blood-brain barrier and maintaining the integrity of tight connections between cells [87].

## Parasite

In recent years, there have been several scattered studies on the role of Annexin A2 in parasites. These studies have investigated various aspects such as calcification in echinococcosis, parasite vaccines, and mechanisms of parasite invasion. It has been found that Annexin A2 plays an auxiliary role in both the infection and extinction of different parasites. Therefore, the understanding of the function of Annexin A2 in parasites is a good choice for developing effective parasite control strategies.

### Echinococcus granulosus

Hydatid disease is a zoonotic illness that poses a significant threat to human health and animal husbandry production. Abundant Annexin A2 is present in the lamina between *Echinococcus granulosus* and the host, which is derived from host granulomatous inflammatory cells [88]. In a study on rats with echinococcosis, treatment with albendazole alone or in combination with matrine alkaloids effectively inhibited *echinococcal* infection and increased Annexin A2 levels [89]. As Annexin A2 is a calcium-binding protein and its levels rise during treatment of echinococcosis, it has been speculated whether Annexin A2 promotes calcification during echinococcosis death. Therefore, the increased level of Annexin A2 may be a defence mechanism against hydatid disease.

### Schistosome

*Schistosomiasis* is a serious disease that poses a significant threat to human health. The identification of new tegument proteins is a key focus of current research in order to enhance our understanding of host-parasite interactions, as well as to provide new potential vaccine antigens against schistosomiasis. Annexin A2 is one such protein that binds to the surface membrane of the tegument in a calcium-dependent manner, and is mainly localized in the tegument of *Schistosoma Mansoni* adult worms [90]. Additionally, another research suggested that Annexin A2 is significantly upregulated during the transition from cercariae to adult parasites. Similarly, it is worth noting that the Annexin A2 ortholog from *Schistosoma Japonicum* was also found to be up-regulated in adult male worms [91]. Therefore, this knowledge confirms the protein's surface exposure and up-regulation in parasites, and its concentration is related to the Schistosoma life cycle, indicating that Annexin A2 may be evaluated as a potential vaccine candidate against *schistosomiasis*.

### Other parasites

*Trichinella Spiralis* has been shown to inhibit the growth of tumour cells, and Deng et al. used *Trichinella Spiralis* infected BALB/C mice to establish an SP2/0 myeloma model. Through the use of suppression subtractive hybridization and other techniques, they identified Annexin A2 as a candidate gene that may be related to tumour growth inhibition in the 180-850 bp sequence [92]. During *Trypanosoma cruzi* infection, the eukaryotic protein ARF-6, which belongs to the ARF family of small GTPases and is widely present, plays a crucial role in regulating membrane trafficking and actin cytoskeleton rearrangements. Specifically, ARF-6 binds to Annexin A2 to promote cell actin recruitment and endocytosis, thereby facilitating the parasite’s invasion [93]. Additionally, Gp35/50 mucins are abundantly expressed on the surface of *Trypanosoma cruzi* G strain metacyclic trypomastigote (MT), which mediate the parasite’s invasion by interacting with host cell Annexin A2 and inducing PTK activation and F-actin recruitment [94]. Furthermore, *Angiostrongylus cantonensis* Acgal-1 induces macrophage apoptosis by interacting with Annexin A2 expressed on the surface of macrophages and activating the JNK signaling pathway. This results in increased expression of apoptotic proteins caspase-3, caspase-9, and Bax, while the expression of the anti-apoptotic protein Bcl-2 is decreased [95]. Overall, Annexin A2 is effective on the host-parasite interaction of various parasites, and its interactions with other proteins and signaling pathways provide important insights into the mechanisms of parasite invasion, inhibition of tumour growth, and apoptosis induction.

Although there have been only a few studies on the topic, the results presented here provide a foundation for further investigation into the pathogenesis of parasite evasion and host pathogenesis. This, in turn, will allow for the development of effective anti-parasite strategies and treatment measures.

## Bacteria

Accumulating evidence suggests that Annexin A2 is helpful to the pathogenesis of several bacterial infections. Gram-negative bacteria, such as *Rickettsia, Escherichia coli, Salmonella Typhimurium, Pseudomonas aeruginosa*, and *Helicobacter pylori*, utilize cell-surface Annexin A2 to mediate adhesion and invasion. In *Rickettsia* infections, Annexin A2 binds to OmpB, a polymeric protein on the outer membrane surface, allowing the bacteria to enter endothelial cells [96,97]. Similarly, in *Escherichia coli* infections, Annexin A2 is recruited to the bacterial adhesion site on the cell surface and interacts with *Espl2*, the *Escherichia coli* secretory protein, enhancing F-actin binding activity, suggesting a potential role in membrane/cytoskeleton reorganization [98,99]. *Espl2* is independent of TIR-mediated actin polymerization, and both proteins bind to Annexin A2 to mediate actin polymerization [99]. Moreover, Annexin A2 has been shown to be involved in reorganizing the actin cytoskeleton during *Salmonella* invasion and serves as a receptor for *Pseudomonas aeruginosa* in mammalian epithelial cells for bacterial internalization into host cells [100,101]. Deficiency in Annexin A2 can impair autophagosome formation and bacterial clearance, increase infiltration of proinflammatory cytokines such as IL-17a and neutrophils, enhance inflammatory responses, and decrease animal survival in *Pseudomonas aeruginosa* infections [102,103]. A similar phenomenon has been found in *Salmonella* infections where Annexin A2 and its ligand S100A10 depletion can block bacterial invasion [100]. However, recent studies have shown that the gram-positive bacteria *Staphylococcus aureus* (*S. aureus*), clumping factor A (ClfA), interacted with Annexin A2, suggesting that ClfA may be responsible for the entry of the bacteria into host cells through a zipper-type mechanism. Annexin A2 may be a possible therapeutic target for bacteria, but further studies are needed to fully understand its role in the pathogenesis of bacterium that rely on the host actin skeleton for disease development.

## Mycoplasma

Some evidence indicated that Annexin A2 is involved in the interaction between *Mycoplasma hyorhinis* (*M. hyorhinis*) and certain tumours, such as gastric cancer and hepatocarcinoma. The phosphorylation of Annexin A2 and its translocation to the host cell membrane is triggered by EGFR. P37, which is the major membrane protein of *M. hyorhinis*, interacts with Annexin A2 to facilitate *M. hyorhinis* infection. This leads to the activation of downstream NF-κB signalling, which promotes the migration of gastric cancer cells [104]. Additionally, *M. hyorhinis* increases the resistance of hepatocellular carcinoma cells to certain nucleoside analogue chemotherapeutic drugs such as cisplatin, gemcitabine, and mitoxantrone through the interaction of p37 and Annexin A2 [105]. The bond between P37 and Annexin A2 can be hindered by a peptide consisting of 30 amino acids located at the N-terminus of Annexin A2, which is referred to as A2PP. Blocking this interaction reduces the phosphorylation of EGFR and Annexin A2, suppressing mycoplasma-induced invasion and migration of gastric cancer cells [106], and improving the sensitivity of hepatocellular carcinoma cells to chemotherapy drugs [105]. According to the above, Annexin A2 both promotes mycoplasma infection, which enhances tumour metastasis, and increases drug resistance in tumour cells.

Studying the potential of A2PP to treat infections caused by *M. gallisepticum* and *M. pneumoniae* is of great value as it can significantly contribute to the development of medicine and agriculture. Furthermore, A2PP has been found to effectively reduce mycoplasma contamination in cell culture. It is noteworthy that A2PP is a more effective and less cytotoxic alternative to commercially available antibiotics commonly used in cell culture [106].

Among other mycoplasmas, *Mycoplasma pneumoniae*’s community-acquired respiratory distress syndrome toxin (CARDS toxin) is an exotoxin that binds to Annexin A2 and promotes its internalization into eukaryotic cells, resulting in cell vacuolation [107]. Similarly, *M. gallisepticum* GroEL protein (HSP60) binds to Annexin A2 and induces PBMC apoptosis by activating the Bax/Bcl2 gene and up-regulating apoptosis-related Caspase protein [108]. Annexin A2 also appears to play a role in *Mycoplasma bovis* invasion into EBL cells by translocating from the cytoplasm to the cell surface and down-regulating inflammatory events [109]. However, the presence of Annexin A2 on the cell surface alone does not appear to be sufficient to mediate mycoplasma infection, as anti-Annexin A2 antibodies do not effectively block mycoplasma infection. It is speculated that Annexin A2 may work in combination with S100A10 to mediate mycoplasma infection. It is noteworthy that research on the role of Annexin A2 in pathogenic mycoplasma remains limited and further efforts are required to better understand this complex interaction.

## Fungal

*Cryptococcus neoformans* is a fungus that is widely distributed around the world and has the potential to cause severe central nervous system infections in individuals who are either immunocompetent or immunocompromised [110]. Previous research on the interaction between Annexin A2 and *Cryptococcus neoformans* has primarily focused on the interaction between fungal cells and endothelial cells. When brain endothelial cells come into contact with and uptake *Cryptococcus neoformans*, Annexin A2 and S100A10 genes are up-regulated. There are two main pathways by which Annexin A2 appears to influence the invasion of *Cryptococcus neoformans* [111]. On the one hand, Annexin A2 phosphorylation induces cofilin phosphorylation in cerebral microvascular endothelial cells, which inhibits the adhesion of *Cryptococcus neoformans* to cells [112]. This differs from the positive regulatory effect of Annexin A2 phosphorylation seen in other pathogens. Additionally, the Src kinase inhibitor, PP2, improves the binding efficiency of *Cryptococcus neoformans* by inhibiting Annexin A2 phosphorylation [112]. On the other hand, Annexin A2 may rely on binding with its partner S100A10 to mediate the transcytosis of *Cryptococcus neoformans* across the endothelium of the brain. The metalloproteinase *Mpr*, a secreted metalloprotease, is also thought to enhance the permeability of the blood-brain barrier by targeting and proteolytically altering surface proteins of brain endothelial cells. *Mp*r interacts with Annexin A2, facilitating the transcellular process of *Cryptococcus neoformans* [113]. During *Cryptococcus neoformans* infection, Annexin A2 loss may reduce the ability of macrophages to control fungal infection, resulting in a significant decrease in phagocytosis and insoluble exocytosis against fungal cells. This may be disadvantageous for *Cryptococcus neoformans* to cross the blood-brain barrier through macrophages [114]. However, the mechanism of Annexin A2 involvement in *Cryptococcus neoformans* invasion is still unclear, and its ligands on *Cryptococcus neoformans* have not been systematically studied. This is currently a hot topic of research.

## Future perspective

Annexin A2 is a crucial protein in pathogen invasion and pathogenesis. It plays a vital role in various processes in the virus life cycle, such as adsorption, penetration, transport, uncoating, biosynthesis, assembly, and release. However, current research has mainly focused on the role of one or a few processes in the virus life cycle, rather than studying the systematic process. Besides, it is necessary to study both Annexin A2 and its ligand, S100A10, in more detail, as they work together in some parts of viruses. Annexin A2 interacts with various microbial proteins, including HCV NS5A and NS5B, HIV glycoprotein, *cytomegalovirus* glycoprotein B, and SADS-CoV M protein. It is, therefore, crucial to study the chemical domain of their interaction. In terms of coronavirus, the study of Annexin A2 is currently limited to autoantigens, and further research is required to investigate the protein’s role in virus infection and pathogenesis. Regarding treatment, various options have been proposed, such as SLPI, anti-Annexin A2 antibody, Annexin A2 inhibitor, and Annexin A2 analog, all of which have shown promise in reducing viral infection. Combining these substances with existing antiviral drugs can have a synergistic effect in antiviral treatment. In parasites, the study of Annexin A2 has focused on its role in adhesion to the host and designing candidate vaccines to enhance host immunity. In bacteria, Annexin A2 promotes adhesion and internalization of Gram-negative bacteria by regulating membrane or cytoskeleton reorganization. Annexin A2 is also involved in HBV, *Mycoplasma genitalium*, and other tumour-causing processes. Therefore, it is essential to improve our understanding of the mechanism by which Annexin A2 is involved in pathogen invasion into host cells and to design targeted drugs against Annexin A2.

## Data Availability

Data sharing is not applicable to this article as no new data were created or analysed in this review.
